# Characterization of hyperkalemic periodic paralysis: a survey of genetically diagnosed individuals

**DOI:** 10.1007/s00415-013-7025-9

**Published:** 2013-07-25

**Authors:** G. Charles, C. Zheng, F. Lehmann-Horn, K. Jurkat-Rott, J. Levitt

**Affiliations:** 1 Department of Family Medicine, Overlook Medical Center, 99 Beauvoir Avenue, Summit, NJ 07902 USA; 2 Department of Internal Medicine, Yale-New Haven Hospital, 20 York Street, New Haven, CT 06510 USA; 3 Institute of Neurophysiology, Ulm University, Albert-Einstein-Allee 11, 89081 Ulm, Germany; 4 Rare Disease Center, Ulm University Medicine, Ulm, Germany; 5 Department of Medical Education, The Icahn School of Medicine at Mount Sinai, 1428 Madison Avenue, New York, NY 10029 USA; 6 Department of Dermatology, Mount Sinai Medical Center, One Gustave L. Levy Place, New York, NY 10029 USA; 7 Periodic Paralysis Association, New York, NY USA

**Keywords:** Periodic paralysis, Hyperkalemia, Flaccid paralysis, Myotonia, Paramyotonia, Stiffness

## Abstract

This exploratory study aims to create an evidence-based comprehensive characterization of hyperkalemic periodic paralysis (hyperPP). HyperPP is a rare genetic disorder that causes episodes of flaccid paralysis. Disease descriptions in the literature are based upon isolated clinical encounters and case reports. We describe the experience of a large cohort of genetically diagnosed individuals with hyperPP. We surveyed genetically characterized individuals age 18 and over to assess disease comorbidities, diagnostic testing, management, and quality of life issues relevant to hyperPP. Myotonia was reported by 55.8 % of subjects and paramyotonia by 45.3 %. There is a relative risk of 3.6 (*p* < 0.0001) for thyroid dysfunction compared to the general population. Twenty-five percent of subjects experienced their sentinel attack in the second decade of life. It took an average of 19.4 years and visits to four physicians to arrive at the diagnosis of hyperPP. In addition to limbs and hands being affected during attacks, 26.1 % of subjects reported their breathing musculature was affected and 62.0 % reported their facial muscles were affected. There was a lifelong trend of increasing attack frequency, which was particularly common during childhood and adolescence. Approximately one-third of individuals experienced progressive myopathy. Permanent muscle weakness was evident and worsened during childhood and after age 40. Those with no chronic treatment regimen have a RR of 2.3 for inadequate disease control compared to those taking long-term medications. This study revealed a multitude of heretofore unidentified characteristics of hyperPP, in addition to providing a different perspective on some previously held notions regarding the condition.

**Electronic supplementary material:**

The online version of this article (doi:10.1007/s00415-013-7025-9) contains supplementary material, which is available to authorized users.

## Introduction

Hyperkalemic periodic paralysis (hyperPP) is an autosomal dominant muscle sodium channelopathy with nearly complete penetrance [1]. Tyler et al. [2] first described the disease in 1951 in their study of a kindred of 7 generations of individuals with clinically typical periodic paralysis in the absence of hypokalemia. In 1957, Gamstorp extensively investigated the condition and named it *adynamia episodica hereditaria* [3]. The name was later changed to hyperkalemic periodic paralysis in light of the provocative effects of potassium intake as well as the rise in serum potassium levels associated with spontaneous attacks [1]. Point mutations in the *SCN4A* gene encoding the alpha subunit of the skeletal muscle voltage-gated sodium channel Nav1.4 lead to defective channel function. This disrupts the normal exchange of ions in skeletal muscles, consequently reducing their ability to contract and resulting in attacks of muscle weakness or paralysis [4, 5]. The condition affects approximately 1 in every 200,000 people [1]. Although typically hereditary, de novo mutations have been reported [6].

The following disease description summarizes the literature on hyperPP to date; our study calls some of this information into question. HyperPP is characterized by attacks of flaccid muscle weakness that are in fact episodic, rather than periodic, in nature. Attacks typically begin in the first decade of life, progressing in frequency until their frequency plateaus in early adulthood. Sometime after midlife, attack frequency declines substantially [7, 8]. Often older individuals experience a chronic progressive myopathy that can cause permanent muscle weakness [8].

Attacks vary in severity, and the same mutation can have variable expressivity within and between families [5]. Attacks can last anywhere from 30 min to several hours [6], typically lasting 1–2 h [9] and infrequently days [10]. Unlike those with hypokalemic periodic paralysis (hypoPP), those with hyperPP infrequently experience generalized flaccid paralysis but rather tend to have focal weakness; the thigh and calf muscles are often affected. Reflexes during an attack may be absent or diminished, and, rarely, the bulbar and respiratory muscles may be affected. Sphincter muscles maintain tonicity during attacks. Sensation is not affected during attacks, although individuals may experience an aura of muscle paresthesia and discomfort prior to the onset of weakness [11].

HyperPP has three clinically distinct manifestations: (1) without myotonia, (2) with clinical or electromyographic (EMG) myotonia, or (3) with paramyotonia congenita (PMC). In all three forms, the course of the paralytic attacks is the same [7, 11]. Electrical myotonia can be demonstrated on EMG in 50–75 % of patients with hyperPP, while less than 20 % manifest clinically [10]. In those with myotonia, i.e., a tonic spasm of muscle [12], the myotonia is often mild and can be provoked with percussion or activity in the face, tongue, forearms, and thenar eminence. The myotonia eases with repetitive activity [11]. From birth onwards, those with PMC experience muscle stiffness that increases with continued activity (paramyotonia) and is cold-induced [1]. Paralytic attacks can occur at any time, though often occur spontaneously in the morning prior to breakfast, last up to an hour, and unpredictably subside [8, 11]. Attacks may be provoked or worsened by anesthesia [5], rest after exercise, potassium loading, cold environments, hunger, emotional stress, glucocorticoids, or pregnancy. During attacks, individuals may be hyperkalemic or normokalemic [4]. The concomitant rise in serum potassium levels can range from upper normal values to those in the cardiotoxic spectrum [6]. After an attack, serum potassium levels may be transiently low due to the elimination of potassium from the kidneys and the reuptake of potassium by muscle [7]. Usually, individuals do not experience cardiac arrhythmias or respiratory insufficiency during attacks [8]. After an attack, individuals may feel pain for up to several days in the involved muscle groups [11]. Between attacks, affected individuals have normal serum potassium levels, normal sensation and muscle stretch reflexes, and normal muscle strength, although they may experience minor myotonia that does not hinder voluntary movement. “Lid lag” secondary to eyelid myotonia may be the only clinical sign present between attacks [8, 11].

The diagnosis of hyperPP is based on clinical grounds, sometimes with the use of provocative tests in cases of diagnostic uncertainty [8]. The diagnosis is suggested by a history of attacks of weakness or paralysis, a positive family history, and the presence of myotonia or paramyotonia. Serum creatine kinase (CK) values may be elevated, and some individuals exhibit calf hypertrophy. The muscles are typically well-developed [6, 11]; however, a large proportion of individuals with hyperPP develop a chronic progressive proximal myopathy as they age [10, 13]. Parenthetically, individuals without interictal myotonia are much more susceptible to developing this progressive myopathy than are individuals with myotonia [1, 8]. Muscle biopsy is non-specific, though will frequently reveal muscle fiber atrophy with vacuoles [5, 11]. Genetic testing is positive in approximately 60 % of individuals who meet clinical diagnostic criteria. An EMG may show myotonic signs, which strongly support the diagnosis, although approximately half of those with the most common mutation show no such signs [8]. Provocative tests, such as the potassium challenge test, pose obvious risks to the patient but may be done to support the diagnosis. The availability of genetic testing and electrophysiologic studies largely obviates the need for such dangerous tests [5, 11].

Prophylactic measures include eating frequent carbohydrate-rich meals and the continuous use of diuretics that reduce serum potassium levels, such thiazides or carbonic anhydrase inhibitors. Equally important is avoidance of potassium-rich foods, medications that raise serum potassium, fasting, strenuous work, and exposure to cold. Mexiletine is beneficial in the management of myotonia. Early in the course of an attack, abortive or attenuating measures include mild exercise, carbohydrate ingestion, and beta-adrenergic agonist inhalation. Severe attacks warrant treatment with intravenous glucose and insulin. Calcium carbonate is used in cases of severe hyperkalemia to stabilize the myocardium to prevent arrhythmia [6, 8, 11]. Patients with hyperPP must avoid depolarizing anesthetics, such as suxamethonium and anticholinesterase agents, as they aggravate myotonia and can interfere with intubation and mechanical ventilation [8].

The present study describes the collective experience of a relatively large cohort of individuals genetically diagnosed with hyperPP. Study objectives include to confirm or refute previously reported descriptions of the disease, to discover new and previously unreported features and associations, and to provide a better understanding of the experience of patients with hyperPP.

## Methods

To characterize the epidemiology, symptoms, diagnostic studies, therapeutic options, and special situations associated with hyperPP, we selected and developed questionnaire items on the basis of a literature search. We then created a survey on SurveyMonkey^®^ (http://www.surveymonkey.com) comprised of multiple choice and short answer questions. To confirm participants indeed had hyperPP, study participants were asked to provide their specific genetic mutation. Only responses of published mutations or unpublished severe amino acid substitutions in typical hyperPP protein areas were included in this report. As the objective of this study was to better characterize a disorder that is not fully characterized, most questions included space for subjects to add comments to their selected responses.

The Mount Sinai School of Medicine’s Institutional Review Board approved the study protocol, and, per protocol, the study was performed according to the ethical standards of the revised 1964 Declaration of Helsinki. Invitations to take the survey were sent via listserv announcements and mailings to members of the following organizations: Periodic Paralysis Association, Periodic Paralysis News Desk, University of Ulm (Germany), Strong Memorial Hospital (University of Rochester), University of Texas Southwestern, University Pierre et Marie Curie (France), and National Hospital for Neurology and Neurosurgery (University College London). All individuals provided their informed consent prior to inclusion in the study. Survey responses were completed either on-line or on paper. Subjects in Germany were provided with a German translation of the survey; their responses were later translated to English for data analysis. The study remained open for approximately 4 months.

Adults (persons 18 years of age or older) with a genetic diagnosis of hyperPP were eligible for this study. For purposes of this study, a genetic diagnosis specifically refers to mutations: A488T, E1702K, F1311V, G1306A, G1456E, I1160V, I1495T, I693T, L1436P, L1489H, L433R, M1360V, M1592V, R1448C, R1448H, S804F, T1313M, T704M, and V1293I, as well as two mutations that have yet to be published.

We collected a total of 137 survey responses. Of these persons, 68.6 % (94/137) provided eligible mutations. Some respondents did not respond to all survey questions. Study calculations for individual questions were typically based only on respondents, e.g., if there were 94 total participants, and only 80 responded to a question, 8 of whom said ‘yes’, then this was reported as a 10 % positive response rate for that question. Absolute values for key study data can be found in the electronic supplementary material. We cannot evaluate how our sample compares to the known hyperPP population considering there is no prior large-scale study of this population. *P* values were calculated on http://graphpad.com/quickcalcs/chisquared1.cfm using chi-square computations with a familywise error rate of 0.05. In the case of multiple comparisons, a Bonferroni correction was made, i.e., the familywise error rate was divided by the number of choices offered to subjects in that question.

## Results and discussion

### Demographics, family history, and comorbidities

The study population was 53.3 % male and 46.7 % female with an age range of 19–84, median age of 46, mode of 48, and mean of 44.9. The majority of subjects (85.7 %) reported residing in Germany. In order to characterize subjects’ degree of disability, we asked whether or not they receive Social Security Disability payments, and 15 % responded that they do. A total of 91.5 % of respondents reported having at least one relative with hyperPP. Of the remaining individuals, most reported multiple relatives with PMC without hyperPP. Those with no family history of hyperPP or PMC appear to have de novo mutations.

To identify comorbidities prevalent in this population, we surveyed subjects for common medical conditions. The most common positive responses included thyroid abnormalities, cardiac arrhythmias, migraines, and high triglycerides/cholesterol. Thyroid dysfunction affects 20.2 % of this population, putting them at a relative risk (RR) of 3.6 (*p* < 0.0001) compared to the general population [14]. Cardiac arrhythmias were reported by 9.6 % of the study population, which was not statistically significant compared to that of the general population (*p* = 0.04; per Bonferroni correction, statistical significance requires *p* < 0.005) [15]. Neither the prevalence of migraine nor hyperlipidemia is increased compared to the general population [16, 17]. Physicians caring for patients with hyperPP should be aware of the increased risk of thyroid dysfunction.

### Diagnosis and symptoms

**Fig. 1:**
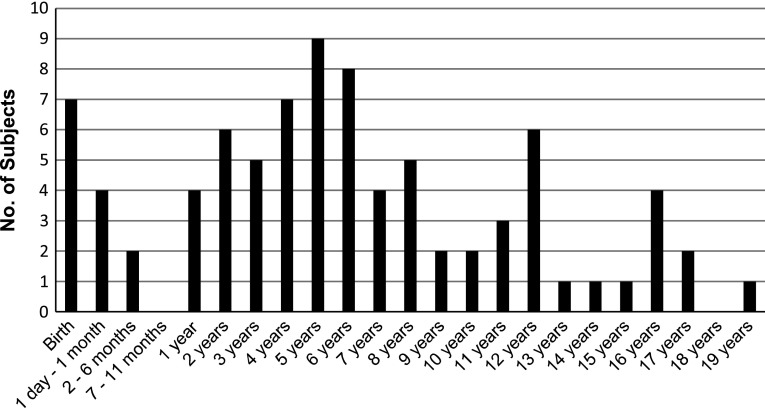
Individuals reported the age at which they experienced their first attack. Sentinel attacks were reported as late as age 19, with 25 % of subjects reporting their first attack at age 10 or older (*No.*, number)

Participants reported their age of first attack as shown in Fig. 1. Twenty-five percent experienced their sentinel attack in the second decade of life, in contrast to the accepted notion that attacks usually begin in the first decade of life [8].

The average diagnostic journey for our cohort from the time of the first onset of symptoms until reaching the correct diagnosis lasted 19.4 years, ranging from an immediate diagnosis due to diagnosed relatives to as long as 65 years. Study participants saw an average of 4 health care professionals, some seeing as many as 10, before obtaining their correct diagnosis. The majority of participants regarded neurologists as the most valuable physicians in reaching their proper diagnosis; several considered either a geneticist or pediatrician as most valuable. Subjects reported undergoing a range of diagnostic studies, most commonly undergoing blood tests, EMGs, and electrocardiograms (EKGs). Surprisingly, 23.4 % of the study population underwent potassium challenges tests, which put them at risk for a severe attack. Before arriving at a diagnosis of hyperPP, subjects were misdiagnosed with conditions such as malingering, conversion disorder, hypoPP, myotonia congenita, and depression. A significant number of these misdiagnoses are psychiatric conditions. Generalizing from this cohort, unless recognized quickly due to family history, the path from attack onset to proper diagnosis is a long one, often taking years, involving a multitude of diagnostic studies and misdiagnoses, and ultimately necessitating a neurologist to clinch the diagnosis.

We surveyed the prevalence of paramyotonia and myotonia in individuals with hyperPP. Overall, 45.3 % of respondents reported paramyotonia, over half of whom reported myotonia as well. One subject reported having trouble letting go of the toothbrush after brushing his teeth and mentioned that this was the first presentation of his daughter’s hyperPP. Myotonia was slightly more prevalent amongst subjects than paramyotonia; overall, 55.8 % of respondents reported symptoms of myotonia, exactly half of whom reported paramyotonia as well. Of note, myotonia presents clinically in markedly more people than the 20 % of individuals reported in prior literature [10]. Parenthetically, 31.4 % of respondents were unsure whether or not they experienced myotonia, and therefore its prevalence may be underrepresented in this survey. Of individuals with myotonia, 37.5 % have experienced progressive myopathy, while of those denying myotonia, 33.3 % have experienced progressive myopathy, which contradicts previous literature reporting that those with myotonia are less susceptible to progressive myopathy [1, 8]. Myotonia may also be exacerbated in pregnancy; as one subject said of being pregnant, “my muscles contract constantly.” Myotonia and paramyotonia appear to affect a larger proportion of individuals with hyperPP than previously recognized.

To identify attack triggers that exist in addition to those previously reported, we surveyed subjects regarding items they have recognized as triggers. Seventy-five point eight percent of subjects reported cold environments, 67.0 % rest after exercise, 47.3 % stress or fatigue, 45.1 % alcohol, 42.9 % hunger, 40.7 % changes in activity level, 35.2 % potassium in food, 35.2 % specific foods or beverages, 35.2 % changes in humidity, 34.1 % extra sleep, 27.9 % (of female respondents) pregnancy, 27.5 % illness of any type, 18.6 % (of female respondents) menstruation, 16.5 % medication, and 14.3 % potassium supplements. The fact that the menstrual cycle and pregnancy were both identified as triggers suggests a role for estrogenic factors in exacerbating hyperPP. Regarding the typical time at which attacks occur, 55.8 % reported the morning, 45.3 % reported during sleep, and 43.0 % reported upon waking, while only 29.1 % identified the afternoon and 19.8 % the evening as typical times for attacks. Sleep shows a propensity to serve as an attack trigger, possibly analogous to rest after exercise on a greater scale.

With respect to prodromal symptoms, the majority of participants reported experiencing symptoms before the onset of a frank attack. During the day prior to an attack, 38.6 % experience symptoms such as fatigue, weakness, irritability, and/or restlessness. Immediately preceding an attack, 85.7 % experience symptoms such as sweating, muscle pain, stiffening, weakness, restlessness, tingling and/or numbness. After such symptoms, most participants experience attack onset within the next 20 min, although some take as long as several hours. Of note, the duration of attack latency from the time of prodrome onset can vary for an individual. In asking subjects to detail their mood before or during attacks, many participants reported being irritable, lethargic, or depressed. Although the experience varies between individuals and between attacks, the general consensus describes one of sweating and/or a change in muscle sensation within the 20 min prior to attack onset.

We surveyed subjects regarding attack frequency and duration. Some subjects commented that their attacks occur less frequently than they otherwise would as they are now medically controlled. Attack frequency spanned the gamut, ranging from 1–3 attacks per month (29.9 %) to once per week (20.7 %) to 2–6 per week (24.1 %). In contrast to prior literature typifying attacks as 1–2 h in duration and infrequently lasting for days, 22.2 % reported typical attacks lasting over 2 days, 21.0 % of subjects reported typical attacks lasting 30 min to an hour, and 13.6 % reported attacks lasting 15–30 min. A total of 22.9 % reported their longest attack lasting for over a week.

**Fig. 2:**
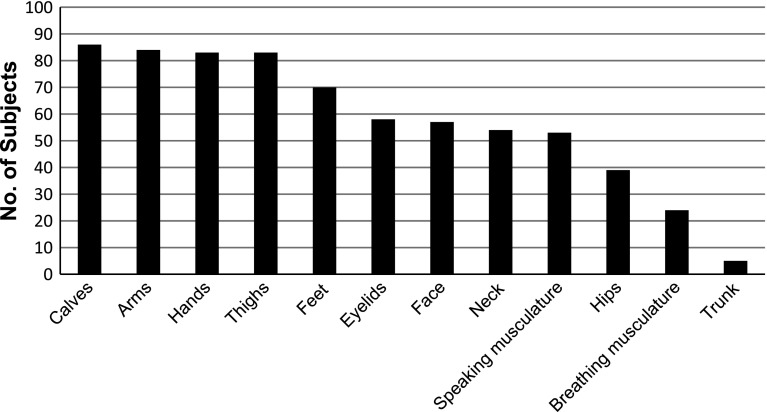
Subjects reported those areas of the body affected during attacks. Arms and hands are just as frequently affected during attacks as thighs and calves. Furthermore, facial and breathing musculature are affected during attacks in a significant number of individuals

Subjects most commonly described their attacks as stiffness followed by weakness, although many described their attacks as some other permutation of weakness and/or stiffness. Regions of the body typically affected by attacks are shown in Fig. 2. The arms and hands are just as frequently affected as the thighs and calves. In contrast to prior literature [11], 26.1 % of subjects reported that their breathing musculature was affected and 62.0 % reported that their face was affected during attacks, 2.2 % reported being unable to speak during a typical attack. Current clinical understanding is that sphincter tone is not altered during attacks [11], however, two subjects reported bowel incontinence and seven reported bladder incontinence during attacks.

The reported severity of attacks varied greatly between individuals. While the largest subset (43.3 %) of respondents described the majority of their attacks as mild (defined as “have only some limitations on mobility, others would notice I am in an attack”), 15.6 % reported their attacks to be either severe (defined as “can speak, cannot move at all, can call for help”) or very severe (defined as “cannot speak, cannot call for help”). The majority of individuals reported mild or no symptoms between attacks. However, 12.4 % reported severe symptoms between attacks that impair their activities of daily living, which stands in contrast to prior literature reporting that “lid lag” secondary to eyelid myotonia may be the only clinical sign present between attacks [8, 11].

After an attack, most subjects reported experiencing various symptoms that have not previously been reported in literature. Most commonly they reported clumsiness, extreme fatigue, weakness, and/or irritability. Worrisome symptoms reported include shortness of breath and palpitations. Reports of muscle pain secondary to attacks were consistent with prior literature: 41.3 % reported muscle pain during attacks and 62.0 % reported muscle pain after attacks; only 26.1 % denied any associated muscle pain.

In order to characterize the course of hyperPP, we surveyed subjects regarding the way in which their attacks have changed over time. Attacks typically increased in frequency during childhood (91.4 %) and teenage years (79.7 %). From age 20–39, the trend was more divided with 52.2 % reporting increasing attack frequency, 30.4 % reporting no change in frequency, and 17.4 % reporting decreased frequency. From age 40–69, 60 % increased, 20 % stayed the same, and 20 % decreased in attack frequency. Several subjects commented that their attack frequency has remained constant throughout their lives. Regarding changes in the severity of muscle weakness during attacks, in individuals under age 20 there was no significant trend. Between ages 20–39, 64.3 % reported improvement, and, from ages 40–69, 55 % reported improvement while 40 % reported worsening weakness. Regarding changes in muscle stiffness during attacks over time, subjects frequently reported worsening stiffness during childhood (85.1 %) and puberty/teenage years (60.9 %). From age 20–39, participants evenly reported no change, worsening, and improvement in muscle stiffness. From age 40–69, 54.5 % reported worsening and 18.2 % reported improvement of muscle stiffness. The majority of respondents noted progressive permanent muscle weakness during childhood (69.2 %), while only a minority of individuals reported such changes during teenage years (33.3 %) and from age 20–39 (30.0 %). This is particularly significant as prior literature reports that permanent muscle weakness is a problem of older adults [8, 13]. In line with prior literature, 84.6 % of respondents age 40 and up reported worsening permanent muscle weakness. The general trend in this study population is one of lifelong increasing attack frequency, which is particularly prevalent during childhood and adolescence, improving weakness during attacks from young adulthood onwards, worsening muscle stiffness during attacks prior to adulthood, and progressive permanent muscle weakness during childhood and after age 40.

In addition to the physical effects of their disease, subjects reported other related hardships. In particular, subjects reported their condition having negative impacts on: work (68.3 %), overall physical health (59.8 %), school (57.3 %), family life (37.8 %), overall mental health (30.5 %), and relationships (25.6 %). Effects on overall physical health reported included weight gain, inability to exercise, and cardiac arrhythmias. Severe attacks have made it difficult if not impossible for some to go to work or to attend school, and moderate attacks can make activities more painful and slow to carry out. Family life is similarly affected by limiting activity with children. Progressive myopathy with muscle dysfunction, reported by 30.4 % of subjects, contributes to such limitations. Ages of those reporting limitations of activity range from 20 to 74 years, with a median of age of 46. Per subjects’ reports, the effects of periodic paralysis extend far outside the chronological boundaries of acute paralytic episodes.

We surveyed subjects regarding their emotional and mental well-being. In thinking about their lives with periodic paralysis, subjects most commonly characterized their overall mental well-being as satisfied, happy, fulfilled, or hopeful. A significant minority reported feeling hopeless, depressed, or lost.

Subjects have experienced difficulties in life secondary to their hyperPP. Some experiences they shared include falling in the street and being almost run over, experiencing social anxiety for fear of an attack, and having to take the help of others or feeling pitied during an attack.

Recommendations offered by subjects for others with hyperPP include:

- Get educated and seek support
- Join the Periodic Paralysis Association to share experiences with and learn from others
- Teach others about the disorder; do not make excuses or lie about it
- Adjust your lifestyle, food, activity, and medications as needed to manage your situation
- Know what works for you—even within the same family, people have different levels of severity
- Do what you have to do to enjoy your life, keep your mind off of the condition as much as possible, and always remembering that it could be worse.

#### Treatment and management

We asked individuals to characterize the extent to which they have established adequate therapeutic regimens. Subjects described their regimens as: “needs improvement” (50.6 %), “mostly controlled” (44.6 %), and “optimal” (4.8 %). Of note, 17.4 % of “mostly controlled” persons denied taking chronic medications, while 61.5 % of those describing their disease status as “needs improvement” denied taking chronic medications. Those not taking chronic medications have a RR of 2.3 (*p* < 0.0001) for experiencing inadequate disease control compared to those taking chronic medications. Therefore, physicians should be proactive in prescribing medications when appropriate. Many participants reported some combination of medications and carbohydrate-rich food as their primary therapeutic regimen. The majority of subjects consume medium-sized meals, and 25.4 % consume smaller-sized meals. Most subjects eat three to five meals a day and carbohydrate-rich snacks every 2–3 h. Carbohydrate sources reported include candy, sugar, bread, and pasta. Subjects avoid alcohol, a range of high potassium foods, diet soda, and cold foods and beverages. Concerning those medications used by “mostly controlled” persons, 21.7 % used hydrochlorothiazide, 21.7 % mexiletine, and 13.0 % flecainide. Individuals tend to use salbutamol or their chronic medications in the acute setting.

We surveyed participants to identify strategies they find effective for mitigating acute attacks outside of their long-term treatment regimens. Common practices include doing gentle exercise, keeping warm, eating sweet foods, encouraging muscle relaxation, and drinking water. Discouragingly, 36.0 % are never or are only occasionally able to abort an attack. Regarding dietary factors that abort or ameliorate acute attacks, subjects recommended a range of specific food and beverage items that are carbohydrate-rich though otherwise seem to reflect personal taste rather than any nutritional trend. Food items reported include chocolate bars, cookies, crackers, Coca-Cola, Gatorade, and sugar. The majority of subjects deny any difference between the impact of solid versus liquid carbohydrates. Subjects reported carbohydrates take effect in anywhere from 10 min to 4–6 h, the majority reporting an effect within an hour. Subjects commented that it is also very important to drink a lot of fluids, such as water or tea. Carbohydrates and hydration appear to be a key part of nonmedical intervention.

Regarding the association between serum potassium levels and attack characteristics, no specific trends were noted. Of those who had their serum potassium levels measured during attacks, the majority reported being hyperkalemic, although several reported being normokalemic and one reported being hypokalemic. Regardless of potassium level, subjects usually felt both stiff and weak during the attacks. No one reported a specific potassium level threshold they knew of at which attacks would occur. Paradoxically, 12.7 % of respondents reported episodes of weakness that improved with potassium intake.

We surveyed individuals regarding the amount of time it took to arrive at their current therapeutic regimens; 48 % of subjects reported taking a decade or longer, 20.0 % 5–10 years, 24.0 % 1–5 years, and 8.0 % under a year, with an average of 11.8 years. Several reported that they are still trying to find an optimal therapeutic regimen.

Some subjects offered creative and practical solutions they devised in order to make their home environments more accommodating to their disease. Many keep at the bedside items they find helpful during an acute attack, such as magnesium, sugar, or a cane. Some have placed exercise equipment in their homes to be able to exercise in a safe environment. Others installed bathroom railings, made their homes wheelchair accessible, added raised toilet seats, or were thoughtful with rug placement. Some require a one-level home, ride an electric scooter, and/or wear an emergency medical alert device. Subjects reported doing low-impact, low-resistance exercises, such as walking, biking, and swimming, to avoid attacks. Incorporating these ideas and accommodations into one’s home and lifestyle may improve safety and help one to deal more effectively with disease-associated limitations.

#### Pregnancy, surgery, anesthesia

We surveyed women regarding their experience during pregnancy. During pregnancy, 91.7 % of women reported experiencing an increase in attack frequency. A total of 80 % reported an improvement in muscle weakness during attacks. Regarding muscle stiffness during attacks, 75 % reported worsening and 25 % improvement. The general experience during pregnancy tends to be increased attack frequency, improved muscle weakness during attacks, and worsened muscle stiffness during attacks.

Subjects were surveyed regarding their experiences with surgery and anesthesia. Non-anesthetic surgical complications were reported by 22.8 %, including complete paralysis after surgery for up to 5 days, hyperthermia, respiratory difficulties, and hypernatremia. One subject reported avoiding complications “because [I] always discussed my condition with the anesthesiologist beforehand and only used advisable anesthetics.” Concerning local anesthesia, 10.5 % reported being adversely effected; specific complications included complete paralysis for hours, respiratory depression, palpitations, and muscle stiffness. Regarding general anesthesia, 29.9 % reported a resulting attack. Specifically, subjects reported severe paralysis involving the muscles of respiration, stiffness, weakness, hyperthermia, and propofol-induced heavy weakness. Unfortunately, the anesthetic agents and the nature of the surgeries were not specified by most subjects. Notwithstanding, individuals with hyperPP are clearly at risk for complications from surgery and anesthetics.

## Conclusion

Findings in our study that add to current knowledge or that counter scientific literature regarding hyperPP include the following points. Progressive myopathy affects approximately one-third of individuals regardless of the presence of concomitant myotonia. The RR of thyroid dysfunction in the study population is 3.6 compared to the general population; the trend toward an increased rate of cardiac arrhythmias in this population was not statistically significant.

A quarter of individuals experienced their sentinel attack in the second decade of life. Symptomatic myotonia was reported by 55.8 % of the study population. Alcohol, changes in humidity, sleep, illness, medication, and menstruation can trigger attacks. Attacks may affect the facial and/or the respiratory muscles; some experience loss of urinary or fecal continence. A total of 12.4 % of subjects reported severe symptoms between attacks that impair their activities of daily living. Individuals may experience permanent muscle weakness starting in childhood. Those with no chronic treatment regimen have a RR of 2.3 for reporting relatively poorer disease control than those taking long-term medications. During acute attacks, over 10 % of individuals may note improvement with potassium intake. Consideration of these factors will allow physicians to develop appropriate and patient-specific management plans to best care for each affected individual.

Limitations of our study include recall bias, over- and under-reporting of symptoms, and the potential mischaracterization of myotonia relative to PMC. Furthermore, subjects’ ability to generate unique responses implies that some of these responses would have been more widely reported if they had been included amongst the multiple choice options. While our study represents among the largest cohorts of genetically characterized hyperPP patients to be queried, 94 patients is still a relatively small number, especially given the possibility of genotype/phenotype subsets within what we know to be hyperPP.

## Electronic supplementary material

Below is the link to the electronic supplementary material.

**Supp 1** Questionnaire provided to study subjects

**Supp 2** Absolute values of study data (* *Jurkat*-*Rott and Lehmann*-*Horn, unpublished*)

Supplementary material 1 (PDF 297 kb)

Supplementary material 2 (PDF 244 kb)
