# Supplementary material for: Characterization of hyperkalemic periodic paralysis: a survey of genetically diagnosed individuals
Source: J Neurol. 2013 Jul 25;260(10):2606–13. doi: 10.1007/s00415-013-7025-9 (PMC3824221; doi:10.1007/s00415-013-7025-9)
Supplement: Supplementary file 1 — Supplementary material 1 (PDF 297 kb) [file 415_2013_7025_MOESM1_ESM.pdf]

Hyperkalemic Periodic Paralysis Management

1. Introduction & Consent Form

Thank you for participating in our survey! Your answers are confidential, and extremely helpful in our research. If you have any questions, please contact us at hyperppsurvey@gmail.com

TITLE OF RESEARCH STUDY:
Title: Hyperkalemic Periodic Paralysis Survey
PRINCIPAL INVESTIGATOR (HEAD RESEARCHER) NAME AND CONTACT INFORMATION:
Name: Jacob Levitt, MD, FAAD
Physical Address: 5 East 98th Street, Fifth Floor, Box 1048, New York, NY 10023
Mailing Address: 5 East 98th Street, Fifth Floor, Box 1048, New York, NY 10023
Phone: (914) 661-1726

WHAT IS A RESEARCH STUDY?
A research study is when scientists try to answer a question about something that we don't know enough about. Participating may not help you or others.
People volunteer to be in a research study. The decision about whether or not to take part is totally up to you. You can also agree to take part now and later change your mind. Whatever you decide is okay. It will not affect your ability to get medical care at Mount Sinai.
Someone will explain this research study to you. Feel free to ask all the questions you want before you decide. Any new information that develops during this research study which might make you change your mind about participating will be given to you promptly.

PURPOSE OF THIS RESEARCH STUDY:
The purpose of this study is to understand more accurately about hyperkalemic periodic paralysis. While the science behind why people get periodic paralysis has become clearer in recent years, there is a lot of information we still do not know about the disease itself. This lack of knowledge results from our inability to collect information about the disease from a lot of people with the disease all at one time. We hope to collect the experiences of many people with hyperkalemic periodic paralysis to find out more about this condition. Specifically, we wish to know if there are other common diseases that people get together with periodic paralysis, what treatments work best for people, what treatments perhaps harm people, what strategies might help reverse or prevent an attack of weakness (such as what foods to eat and when to eat them), if periodic paralysis results in disability for people, difficulties in obtaining a diagnosis, unrecognized triggers and symptoms, and experiences with anesthesia and pregnancy.
You may qualify to take part in this research study because you are diagnosed with hyperkalemic periodic paralysis by genetic testing. If you are a member of the Periodic Paralysis Association, your membership will not be affected by your participation or lack of participation.
Funds for conducting this research are provided by Mount Sinai School of Medicine.

LENGTH OF TIME AND NUMBER OF PEOPLE EXPECTED TO PARTICIPATE
Your participation in this research study is expected to last about one hour (or however long it takes you to complete the survey). The survey need not be completed in one sitting.
The number of people expected to take part in this research study at this site is about 30.

DESCRIPTION OF WHAT'S INVOLVED:
If you agree to participate in this research study, the following information describes what may be involved.
• You will be asked to complete a detailed survey about your general health as it pertains to your hyperkalemic periodic paralysis. This is part of the research study and not part of your general health care.
• You may receive a phone call to clarify the results you submit from one of the three investigators on the study. We hope to limit these interactions to a minimum out of respect for your time.
• The survey will be administered online through Survey Monkey, an online survey program. Data analysis will occur at The Mount Sinai School of Medicine.
• The research will be conducted through the months of July, August, and September, and results analyzed in the following two months after that. We hope to have preliminary results by the PPA Bienniel Conference in November, 2011.

***1. By selecting "I agree," you are consenting to the above and indicating that you would like to continue.**

◯ I agree

◯ I do not agree

2.

3. Consent Form - Page 2

YOUR RESPONSIBILITIES IF YOU TAKE PART IN THIS RESEARCH:
If you decide to take part in this research study you will be responsible for completing the survey in its entirety in a timely manner (we hope within one month of beginning the survey). One reason for delay might be that you request medical information from an old provider to be able to answer questions more accurately.

COSTS OR PAYMENTS THAT MAY RESULT FROM PARTICIPATION:
This research will result in no cost to you. You will not be paid to participate in this study.

POSSIBLE BENEFITS:
It is important to know that you may not get any benefit from taking part in this research. Others may not benefit either. However, possible benefits may be better clarification of your disease and general health, which may enable you to explain your condition better to health care providers. You may also bring to light harmful or beneficial trends in your lifestyle as part of completing this survey. Others may benefit from this survey by sharing of successful management tips and validating life experiences that others may have thought were unique to themselves alone. Better defining the disease will allow doctors to diagnose and treat the disease more efficiently as well as defend disability claims more easily when warranted.

REASONABLY FORESEEABLE RISKS AND DISCOMFORTS:
Risks of this survey study are minimal. There always exists the potential for loss of private information; however, there are procedures in place to minimize this risk.

OTHER POSSIBLE OPTIONS TO CONSIDER:
You may decide not to take part in this research study without any penalty. The choice is totally up to you.

IN CASE OF INJURY DURING THIS RESEARCH STUDY:
If you believe that you have suffered an injury related to this research as a participant in this study, you should contact the Principal Investigator.

ENDING PARTICIPATION IN THE RESEARCH STUDY:
You may stop taking part in this research study at any time without any penalty. This will not affect your ability to receive medical care at Mount Sinai or to receive any benefits to which you are otherwise entitled.
If you decide to stop being in the research study, please contact the Principal Investigator or the research staff. At that point, we will exclude your data from the data analyses. No new information will be collected from you.
You may also withdraw your permission for the use and disclosure of any of your protected information for research, but you must do so in writing to the Principal Investigator at the address on the first page. Even if you withdraw your permission, the Principal Investigator for the research study may still use the information that was already collected if that information is necessary to complete the research study. Your health information may still be used or shared after you withdraw your authorization if you should have an adverse event (a bad effect) from participating in the research study.
Withdrawal without your consent: The study doctor or the institution may stop your involvement in this research study at any time without your consent. This may be because the research study is being stopped, the instructions of the study

team have not been followed, the investigator believes it is in your best interest, or for any other reason. If specimens or data have been stored as part of the research study, they too can be destroyed without your consent.

***1. By selecting "I agree," you are consenting to the above and indicating that you would like to continue.**

- ◯ I agree
- ◯ I do not agree

4. Consent Form - Page 3

CONTACT PERSON(S):
If you have any questions, concerns, or complaints at any time about this research, or you think the research has hurt you, please contact the office of the research team and/or the Principal Investigator at phone number (914) 661-1726.
This research has been reviewed and approved by an Institutional Review Board. You may reach a representative of the Program for Protection of Human Subjects at Mount Sinai School of Medicine at telephone number (212) 824-8200 during standard work hours for any of the following reasons:

• Your questions, concerns, or complaints are not being answered by the research team.
• You cannot reach the research team.
• You are not comfortable talking to the research team.
• You have questions about your rights as a research subject.
• You want to get information or provide input about this research.

DISCLOSURE OF FINANCIAL INTERESTS:
None.

MAINTAINING CONFIDENTIALITY – HIPAA AUTHORIZATION:
As you take part in this research project it will be necessary for the research team and others to use and share some of your private protected health information. Consistent with the federal Health Insurance Portability and Accountability Act (HIPAA), we are asking your permission to receive, use and share that information.

What protected health information is collected and used in this study, and might also be disclosed (shared) with others?
As part of this research project, the researchers will collect your name, email, telephone number, address, and date of birth.
During the study the researchers will gather information by taking a medical history (includes current and past medications or therapies, illnesses, conditions or symptoms, family medical history, allergies, etc.).

Why is your protected health information being used?
Your personal contact information is important to be able to contact you during the study. Your health information and the results of any tests and procedures being collected as part of this research study will be used for the purpose of this study as explained earlier in this consent form. The results of this study could be published or presented at scientific meetings, lectures, or other events, but would not include any information that would let others know who you are, unless you give separate permission to do so.
The research team and other authorized members of The Mount Sinai Hospital and Mount Sinai School of Medicine (together, “Mount Sinai”) workforce may use and share your information to ensure that the research meets legal, institutional or accreditation requirements. For example, the Mount Sinai School of Medicine Program for the Protection of Human Subjects is responsible for overseeing research on human subjects, and may need to see your information. If you receive any payments for taking part in this study, the Mount Sinai Medical Center Finance Department may need your name, address, social security number, payment amount, and related information for tax reporting purposes. If the research team uncovers abuse, neglect, or reportable diseases, this information may be disclosed to appropriate authorities.

***1. By selecting "I agree," you are consenting to the above and indicating that you would like to continue.**

- ◯ I agree
- ◯ I do not agree

5. Consent Form - Page 4

Who, outside Mount Sinai, might receive your protected health information?
In all disclosures outside of Mount Sinai, you will not be identified by name, address, telephone number, or any other direct personal identifier unless disclosure of the direct identifier is required by law. Some records and information disclosed may be identified with a unique code number. The Principal Investigator will ensure that the key to the code will be kept in a locked file, or will be securely stored electronically. The code will not be used to link the information back to you without your permission, unless the law requires it, or rarely if the Institutional Review Board allows it after determining that there would be minimal risk to your privacy. It is possible that a sponsor or their representatives, a data coordinating office, a contract research organization, will come to inspect your records. Even if those records are identifiable when inspected, the information leaving the institution will be stripped of direct identifiers. Additionally, the monitors, auditors, the IRB, the Food and Drug Administration, The United States Department of Health and Human Services, and the Office of
Human Research Protection. will be granted direct access to your medical records for verification of the research procedures and data. By signing this document you are authorizing this access. We may publish the results of this research. However, we will keep your name and other identifying information confidential.

For how long will Mount Sinai be able to use or disclose your protected health information?
Your authorization for use of your protected health information for this specific study does not expire.

Will you be able to access your records?
During your participation in this study, you will have access to your medical record and any study information that is part of that record. The investigator is not required to release to you research information that is not part of your medical record.

Do you need to give us permission to obtain, use or share your health information?
NO! If you decide not to let us obtain, use or share your health information you should not sign this form, and you will not be allowed to volunteer in the research study. If you do not sign, it will not affect your treatment, payment or enrollment in any health plans or affect your eligibility for benefits.

***1. By selecting "I agree," you are consenting to the above and indicating that you would like to continue.**

- ◯ I agree
- ◯ I do not agree.

6. Consent Form - Page 5

Can you change your mind?
You may withdraw your permission for the use and disclosure of any of your protected information for research, but you must do so in writing to the Principal Investigator at the address on the first page. Even if you withdraw your permission, the Principal Investigator for the research study may still use your protected information that was already collected if that information is necessary to complete the study. Your health information may still be used or shared after you withdraw

your authorization if you should have an adverse event (a bad effect) from being in the study. If you withdraw your permission to use your protected health information for research that means you will also be withdrawn from the research study, but standard medical care and any other benefits to which you are entitled will not be affected. You can also tell us you want to withdraw from the research study at any time without canceling the Authorization to use your data.

It is important for you to understand that once information is disclosed to others outside Mount Sinai, the information may be re-disclosed and will no longer be covered by the federal privacy protection regulations. However, even if your information will no longer be protected by federal regulations, where possible, Mount Sinai has entered into agreements with those who will receive your information to continue to protect your confidentiality.

If as part of this research project your medical records are being reviewed, or a medical history is being taken, it is possible that HIV-related information may be revealed to the researchers. If that is the case, the information in the following box concerns you. If this research does not involve any review of medical records or questions about your medical history or conditions, then the following section may be ignored.

Notice Concerning HIV-Related Information

If you are authorizing the release of HIV-related information, you should be aware that the recipient(s) is (are) prohibited from re-disclosing any HIV-related information without your authorization unless permitted to do so under federal or state law. You also have a right to request a list of people who may receive or use your HIV-related information without authorization. If you experience discrimination because of the release or disclosure of HIV-related information, you may contact the New York State Division of Human Rights at (212) 480-2522 or the New York City Commission of Human Rights at (212) 306-5070. These agencies are responsible for protecting your rights.

*** 1. By selecting "I agree," you are consenting to the above and indicating that you would like to continue.**

- ◯ I agree
- ◯ I do not agree

7. General Information

1. Please provide an e-mail address at which we may contact you.

2. Please enter your state/province and country of residence:

State/Province:

Country:

3. Please select your year of birth.

4. Please select your diagnosis below.

- ◯ Hyperkalemic periodic paralysis only
- ◯ Hyperkalemic periodic paralysis with paramyotonia congenita

Comments

5. Please select your SCN4A mutation, or that of your family member. If your mutation is known but not listed, please specify in the box provided. If your mutation is not known (i.e., you are not genetically characterized), please do not proceed with this survey.

Note: There are several ways of writing down a specific mutation. We have provided several synonyms in each answer choice; for example, T704M = Thr704Met = C2188T.

6. Does your muscle stiffness increase with continued activity (paramyotonia)?

- ◯ Yes
- ◯ No
- ◯ Unsure

7. Gender:

- ◯ Male
- ◯ Female

8. Height:

	Inches	Centimeters
Please fill in only one column.		

9. Weight:

	Pounds	Kilograms
Please fill in only one column.		

10. Please list your occupations over the past 10 years in chronological order, starting with the most recent first:

Hyperkalemic Periodic Paralysis Management

11. Do you receive Social Security disability? If yes, please indicate the grounds for which you are receving it in the box below.

- ◯ No
- ◯ Yes

Comments:

12. Do any family members also have hyperkalemic periodic paralysis? Please check all that apply.

- ☐ None
- ☐ Maternal grandparent
- ☐ Paternal grandparent
- ☐ Aunt/Uncle
- ☐ Parent
- ☐ Sibling
- ☐ Child
- ☐ Grandchild
- ☐ Other (please specify)

13. Please list which family members, if any, have been affected by hyperkalemic periodic paralysis or paramyotonia congenita.

Hyperkalemic Periodic Paralysis	
Paramyotonia Congenita	
Hypokalemic Periodic Paralysis	
Myotonias	
Other (please specify)	

14. Do you have any other illnesses? Please check all that apply and elaborate on your checked medical condition/s in the box below.

- [ ] Diabetes type 1
- [ ] Diabetes type 2
- [ ] High triglycerides/cholesterol
- [ ] High blood pressure
- [ ] Migraines
- [ ] Coronary artery disease
- [ ] Heart rhythm abnormalities
- [ ] ADD/ADHD
- [ ] Thyroid problems
- [ ] Kidney problems
- [ ] Other (please specify)

8. Diagnosis & Symptoms

1. Age of first attack:

2. What symptoms caused you to seek medical treatment for your condition before you knew your diagnosis?

3. How long did it take to arrive at the correct diagnosis of hyperkalemic periodic paralysis?

4. How many different doctors did you visit before you arrived at your diagnosis?

5. Which specialists did you see leading up to your diagnosis? Please rank the value of each physician seen (1 = least valuable, 5 = most valuable).

	1	2	3	4	5	Did not see this specialist
Neurologist	○	○	○	○	○	○
Endocrinologist	○	○	○	○	○	○
Psychiatrist	○	○	○	○	○	○
Nephrologist	○	○	○	○	○	○
Homeopathic or Alternative Medicine Specialists	○	○	○	○	○	○
Physiatrist	○	○	○	○	○	○

Other (please specify type of physician and value)

6. What tests did you have to arrive at your diagnosis? Please check all that apply. If possible, please call your doctor to clarify if not sure.

- ☐ Muscle biopsy
- ☐ EMG (electromyogram)
- ☐ Nerve conduction study
- ☐ CMAP (compound muscle action potential)
- ☐ Creatine kinase (CK)
- ☐ EKG (electrocardiogram)
- ☐ Genetic testing
- ☐ Potassium challenge test
- ☐ Fasting challenge test (i.e., inducing an attack by not eating)
- ☐ Blood tests
- ☐ Urine tests
- ☐ Other (please specify)

7. Please list various misdiagnoses you were given prior to genetic confirmation of hyperkalemic periodic paralysis.

- [ ] Conversion disorder
- [ ] Myasthenia gravis
- [ ] Multiple sclerosis
- [ ] Hypokalemic periodic paralysis
- [ ] Myotonia congenita
- [ ] Andersen-Tawil syndrome (formerly Andersen syndrome)
- [ ] Depression
- [ ] Diuretic abuse
- [ ] Faking it
- [ ] Malingering (i.e., faking it to get disability or other gain)
- [ ] Other (please specify)

8. Do you notice any signs/symptoms in the preceding day or so before the onset of an attack (e.g., dark circles under the eyes, irritability, etc.)?

9. What symptoms do you experience immediately before an attack?

10. Typically how long after recognizing warning signs/symptoms do you experience a full attack?

Other (please specify)

Hyperkalemic Periodic Paralysis Management

11. When you get attacks, they are (check all that apply):

- ☐ Weakness only
- ☐ Stiffness only
- ☐ Stiffness followed by weakness
- ☐ Weakness followed by stiffness
- ☐ Most often characterized by stiffness
- ☐ Most often characterized by weakness

12. When you get attacks of weakness, are they typically:

- ○ Total body, regardless of prior activity
- ○ Partial body, regardless of prior activity
- ○ Only muscles used prior to the attack

13. Which parts of the body are affected by the muscle stiffness or weakness? Please check all that apply.

- ☐ speaking musculature
- ☐ breathing musculature
- ☐ face
- ☐ eyelids
- ☐ neck
- ☐ arms
- ☐ hands
- ☐ hips
- ☐ thigh
- ☐ calf
- ☐ feet
- ☐ Other (please specify)

14. Most of my attacks are:

- ◯ Very severe – cannot speak, cannot call for help
- ◯ Severe – can speak, cannot move at all, can call for help
- ◯ Moderate – have some movement, can self-medicate
- ◯ Mild – have only some limitations on mobility, others would notice I am in an attack
- ◯ Very mild – barely noticeable to others, few limitations

15. What activities are you still able to perform when in the attack of most common severity? (Please check all that apply)

- ◯ Speak
- ◯ Hold a pencil
- ◯ Get out of bed
- ◯ Walk
- ◯ Walk up stairs without assistance/banister
- ◯ Jump up and down comfortably

16. How severe are your symptoms between attacks?

- ◯ No symptoms
- ◯ Mild
- ◯ Moderate
- ◯ Severe, impairs activities of daily living

17. If you have symptoms between attacks, what are they typically characterized by?

- ◯ No symptoms between attacks
- ◯ Muscle weakness
- ◯ Muscle stiffness
- ◯ Both

18. Do you experience muscle pain during and/or after attacks of paralysis? Please check all that apply.

- ☐ Yes - During attacks
- ☐ Yes - After attacks
- ☐ No

19. What other symptoms do you experience after an attack?

- [ ] No symptoms
- [ ] Muscle pain
- [ ] Extreme fatigue
- [ ] Thirst
- [ ] Shortness of breath (either due to anxiety or to the episode itself)
- [ ] Palpitations
- [ ] Clumsiness
- [ ] Irritability
- [ ] Mental dullness
- [ ] Other (please specify)

20. Has your illness negatively affected any of the following? Please check all that apply.

- [ ] Overall mental health
- [ ] Overall physical health (e.g., weight gain, heart disease, depression, etc. - please specify in comments box below).
- [ ] Family life
- [ ] Relationships
- [ ] Work
- [ ] School
- [ ] Other (please specify)

21. Do you feel that your weight has been affected by your hyperkalemic periodic paralysis?

- ( ) Yes - weight gain
- ( ) Yes - weight loss
- ( ) No

Comments

22. If applicable, what aspects of your condition do you feel have affected your weight?

- [ ] More carbohydrate intake
- [ ] Less physical activity
- [ ] Other (please specify)

23. How frequently do you have attacks?

24. What is the duration of a typical attack?

25. What was the duration of your longest attack?

26. What triggers your attacks? Please check all that apply.

- [ ] Rest after exercise
- [ ] Hunger
- [ ] Specific foods or beverages
- [ ] Alcohol
- [ ] Cold
- [ ] Change in humidity
- [ ] Sickness
- [ ] Extra sleep
- [ ] Stress/fatigue
- [ ] Medications
- [ ] Menstrual cycle
- [ ] Pregnancy
- [ ] Changes in level of activity
- [ ] Potassium in food
- [ ] Potassium supplements (prescription or over-the-counter)
- [ ] Other (please specify)

27. Typical time of day attack occurs (check all that apply):

- [ ] Morning
- [ ] Afternoon
- [ ] Evening
- [ ] Night
- [ ] Upon waking
- [ ] During sleep (please specify time of day, e.g. nap, middle of the night, etc. in "Other" box)
- [ ] Other (please specify)

28. When exposed to cold temperatures, do your muscles slowly relax after voluntary contraction (myotonia)?

- ◯ Yes
- ◯ No
- ◯ Unsure

29. Have you experienced progressive myopathy (muscle dysfunction) or permanent weakness?

- ◯ Yes
- ◯ No
- ◯ Unsure

30. What additional symptoms do you experience in association with your attacks? Please check all that apply.

- [ ] Numbness/tingling
- [ ] Sweating
- [ ] Palpitations
- [ ] Shortness of breath/Difficulty breathing
- [ ] Loss of bladder control
- [ ] Loss of bowel control
- [ ] Other (please specify)

31. While we have attempted to characterize your attacks in the preceding questions, sometimes a free description better describes them. Please describe your typical attack(s) in the space provided.

32. If you feel you have unique attacks that you wish to describe here, please do so.

9. Treatment & Management

1. What medications have you used in your lifetime to treat your attacks? Please check all that apply.

	Hydrochlorothiazide	Diamox (Acetazolamide)	Daranide (Dichlorphenamide)	Lasix (Furosemide)	Mexitil (Mexiletine)	Salbutamol	IV calcium gluconate	Glucose tablets
Chronic (to prevent attacks)	☐	☐	☐	☐	☐	☐	☐	☐
Acute (to treat attacks)	☐	☐	☐	☐	☐	☐	☐	☐

Other (please specify if chronic or acute)

2. Other than the medications in Question 1, what interventions do you undertake to treat an acute attack?

- ◯ Gentle exercise
- ◯ Keeping warm
- ◯ Sugary food (please specify in "Other" box)
- ◯ Other (please specify)

3. How often are you able to abort an acute attack (stop it from progressing further) by the above treatments and interventions?

- ◯ All of the time
- ◯ Most of the time
- ◯ Some of the time
- ◯ Occasionally
- ◯ Never

Comments

4. What level of control do you feel you have achieved with your current regimen of therapy?

- ◯ Optimal
- ◯ Mostly controlled
- ◯ Needs improvement

5. What medication, combination of medications, or foods have you found to achieve optimal control?

6. What medications, foods, or other items do you have on hand to treat an acute attack? Please specify for each.

Medications

Foods

Other

7. For each choice in Question 5, how long does it take to work?

Medications

Foods

Other

8. What foods do you find helpful for your condition? In the comments box, please indicate your preferences, such as which specific candies you find most helpful, as well as any other foods not listed here.

- [ ] Non-diet soda
- [ ] Sweetened iced tea
- [ ] Unsweetened tea
- [ ] Fruit juice
- [ ] Vegetable juice
- [ ] Candy
- [ ] Cereal
- [ ] Oatmeal
- [ ] Pasta
- [ ] Bread
- [ ] White potatoes
- [ ] Sweet potatoes
- [ ] Sugar
- [ ] Yogurt
- [ ] Ice cream
- [ ] Comments

9. Do whole grain carbohydrates affect you differently than refined carbohydrates? If so, please describe the different effects.

- ( ) I don't know
- ( ) I don't eat whole grains
- ( ) No
- ( ) Yes

10. What foods do you avoid due to your condition? Included below are high potassium foods that may trigger attacks (http://www.hkpp.org/faq/hyperkalemic_periodic_paralysis.html).

- [ ] Cantaloupes
- [ ] Apricots
- [ ] Dried figs
- [ ] Kiwis
- [ ] Peaches
- [ ] Raisins
- [ ] Bananas
- [ ] Prunes
- [ ] Fruit juice
- [ ] Artichoke
- [ ] Parsnip
- [ ] Potato
- [ ] Pumpkin
- [ ] Spinach
- [ ] Broccoli
- [ ] Brussels sprouts
- [ ] Cauliflower
- [ ] Tomato juice or tomato-based juices
- [ ] Other vegetable juices
- [ ] Lentils and beans
- [ ] Nuts
- [ ] Peanut butter
- [ ] Chocolate
- [ ] Alcohol
- [ ] Diet soda
- [ ] Cold foods
- [ ] Cold beverages
- [ ] Other (please specify)

11. How frequently do you eat regular meals as a result of your condition?

12. How frequently do you eat carbohydrate-rich snacks throughout the day?

13. Have you noticed that certain forms of sweets work more quickly? If so, which?

- ◯ Liquid sweets (e.g., Coke) work more quickly
- ◯ Solid sweets (e.g., candy) work more quickly
- ◯ I have not noticed/Equal

Comments (if you have a special way of taking sweets e.g., under the tongue, please note here)

14. What quantity of food do you prefer to eat at a time due to its effect on your disease? Please comment on what effect eating other quantities has on you.

- ◯ Smaller quantities
- ◯ Medium quantities
- ◯ Larger quantities

Comments

15. How long did it take for you to arrive at your current regimen of therapy?

Other (please specify)

16. Please list all medications (with dosages, if possible) that you have tried in chronological order.

17. We tried to tease out what medications you tried and each dose. In as much detail as you can recall, please describe how your regimen evolved early on from your current regimen.

18. Have you found that certain medications do not work? If yes, please specify.

- ◯ No
- ◯ Yes (please specify)

19. Are there medications that trigger attacks or worsen your general condition? Please specify.

- ◯ No
- ◯ Yes (please specify)

Hyperkalemic Periodic Paralysis Management

20. What side effects do you experience from your medication? Please check all that apply.

- [ ] None
- [ ] Palpitations
- [ ] Gastric irritation
- [ ] Cognitive dulling
- [ ] Kidney stones
- [ ] Excessive urination
- [ ] Other (please specify)

21. The next few questions (21-25) attempt to elucidate how your disease has changed over time.

How has the frequency of your attacks changed over time?

	Worsened	Improved	Stayed the Same	N/A
Childhood	○	○	○	○
Puberty/Teenage Years	○	○	○	○
age 20-39	○	○	○	○
age 40-69	○	○	○	○
age 70+	○	○	○	○
Pregnancy	○	○	○	○
Menopause	○	○	○	○

Comments

22. How has your muscle weakness during attacks changed over time?

	Worsened	Improved	Stayed the Same	N/A
Childhood	○	○	○	○
Puberty/Teenage Years	○	○	○	○
age 20-39	○	○	○	○
age 40-69	○	○	○	○
age 70+	○	○	○	○
Pregnancy	○	○	○	○
Menopause	○	○	○	○

Comments

23. How has your muscle stiffness during attacks changed over time?

	Worsened	Improved	Stayed the Same	N/A
Childhood	◯	◯	◯	◯
Puberty/Teenage Years	◯	◯	◯	◯
age 20-39	◯	◯	◯	◯
age 40-69	◯	◯	◯	◯
age 70+	◯	◯	◯	◯
Pregnancy	◯	◯	◯	◯
Menopause	◯	◯	◯	◯

Comments

24. If applicable, have you noticed any changes in permanent muscle weakness over time?

	Worsened	Improved	Stayed the Same	N/A
Childhood	◯	◯	◯	◯
Puberty/Teenage Years	◯	◯	◯	◯
age 20-39	◯	◯	◯	◯
age 40-69	◯	◯	◯	◯
age 70+	◯	◯	◯	◯
Pregnancy	◯	◯	◯	◯
Menopause	◯	◯	◯	◯

Comments

25. Over the years, if you feel your symptoms have additionally changed in other ways, please describe.

26. What accommodations do you make in your home environment to help manage your disease?

- [ ] None
- [ ] Rug placement
- [ ] Items kept within reach near bed (e.g. water, carbohydrates, phone, medication - please specify in "Other" box)
- [ ] Emergency access button or necklace
- [ ] Exercise equipment
- [ ] Wheelchair accessibility
- [ ] Bathroom railings
- [ ] Other (please specify)

27. What do you find is an effective way to exercise while avoiding an attack?

- [ ] Walking
- [ ] Yoga
- [ ] Pilates
- [ ] Tai chi
- [ ] Karate
- [ ] Swimming
- [ ] Biking - indoors
- [ ] Biking - outdoors
- [ ] Jogging - treadmill
- [ ] Jogging - outdoors
- [ ] Running - treadmill
- [ ] Running - outdoors
- [ ] Sprints - treadmill
- [ ] Sprints - outdoors
- [ ] Aerobic exercise
- [ ] Other (please specify)

28. What do you do to avoid the cold?

- ( ) I do not need to avoid the cold
- ( ) Warm clothing
- ( ) Heated rooms
- ( ) Avoid the outdoors
- ( ) Electric blanket
- ( ) Other (please specify)

29. Literature suggests that attacks occur more frequently on holidays due to people eating richer meals, sleeping in later, and changing normal activity levels. Is this the case for you?

- ◯ Yes, this is the case for me.
- ◯ No, I do not experience an increase in attacks over holidays.

If yes, how do you manage? If no, what if any preventive measures do you take?

30. If your potassium has ever been measured during an attack, what was the result and how did you feel? If you are unsure or your potassium has not been checked, click "N/A."

	Weak	Stiff	Both	N/A
Hyperkalemic	◯	◯	◯	◯
Hypokalemic	◯	◯	◯	◯
Normal	◯	◯	◯	◯

31. Is there a potassium threshold above which attacks are certain to occur?

- ◯ I don't know
- ◯ No
- ◯ Yes (please specify)

32. Do you have a glucometer at home?

- ◯ No
- ◯ Yes - I don't use it/I don't keep track of levels
- ◯ Yes - I do not see a correlation between glucose levels and attacks
- ◯ Yes - attacks tend to occur below a certain level (please specify):

33. Some people report being affected by hyperkalemic periodic paralysis but also report episodes of weakness associated with low blood potassium that is improved with potassium intake. Is this the case with you?

- ◯ No
- ◯ Yes - please specify your form of potassium intake (foods, pills, etc.)

10. Special Situations & Conclusion

1. If you have ever been pregnant, what special precautions did you take during pregnancy?

2. If applicable, how did attacks change during pregnancy?

	More	Less	Same
Severity	◯	◯	◯
Frequency	◯	◯	◯

3. If you have ever had surgery, have you ever had a complication related to periodic paralysis (excluding anesthesia)?

◯ I have never had surgery

◯ I have never had a complication from surgery related to periodic paralysis

◯ I have had complications from surgery related to periodic paralysis (please specify)

4. If you have ever had local anesthesia, did it trigger an attack?

◯ I have never had local anesthesia.

◯ Local anesthesia did not trigger an attack

◯ Yes (please list the medication and describe the reaction)

5. If you have ever had general anesthesia, did it trigger an attack?

◯ I have never had general anesthesia

◯ General anesthesia did not trigger an attack

◯ Yes (please list the medication and describe the reaction)

6. The following series of questions seeks to define the effect of attacks on mood and overall mental well-being. Just before or during attacks, my overall mood is:

- [ ] Irritable
- [ ] Depressed
- [ ] Lethargic
- [ ] Happy
- [ ] Other (please specify)

7. Before or during an attack, do family members or others notice:

- [ ] No change in mood
- [ ] Increased irritability
- [ ] Increased lethargy
- [ ] Depressed mood
- [ ] Other (please specify)

8. In thinking about your life with periodic paralysis, would you characterize your overall mental well-being as:

- [ ] Depressed
- [ ] Satisfied
- [ ] Happy
- [ ] Fulfilled
- [ ] Lost
- [ ] Hopeless
- [ ] Hopeful
- [ ] Other (please specify)

9. Please share any extreme stories or difficulties you experienced due to hyperkalemic periodic paralysis.

Hyperkalemic Periodic Paralysis Management

10. What recommendations do you have for others with hyperkalemic periodic paralysis?

11. Please let us know of any additional comments you might have here.
