# Supplementary material for: Characterization of hyperkalemic periodic paralysis: a survey of genetically diagnosed individuals
Source: J Neurol. 2013 Jul 25;260(10):2606–13. doi: 10.1007/s00415-013-7025-9 (PMC3824221; doi:10.1007/s00415-013-7025-9)
Supplement: Supplementary file 2 — Supplementary material 2 (PDF 244 kb) [file 415_2013_7025_MOESM2_ESM.pdf]

**Gender**			
Respondents	92		
Male	49		
Female	43		
**Mutations (all other mutations were represented by ≤ 3 subjects)**			
Respondents	94		
T704M	27		
M1592V	19		
I692M*	10		
R1448H	7		
T1313M	5		
**Comorbid Conditions**			
Thyroid	19		
High blood pressure	19		
Arrhythmias	9		
Migraines	9		
High triglycerides/cholesterol	8		
Kidney problems	5		
Diabetes, type 2	4		
Coronary artery disease	3		
Attention deficit disorder/Attention deficit hyperactivity disorder	1		
Diabetes, type 1	0		
**Age of First Attack**			
Respondents	84		
Age 10-19	21		
**Diagnostic studies underwent prior to diagnosis of hyperPP**			
Blood tests	63		
EMG	46		
EKG	30		
Muscle biopsies	26		
Urine tests	25		
Nerve conduction study	22		

Potassium challenge	22		
Creatine kinase	17		
Fasting challenge	16		
Compound muscle action potential test	8		
Ice water test	2		
**Concomitant Paramyotonia and/or Myotonia**			
Respondents	86		
Paramyotonia	39		
Paramyotonia + myotonia	24		
Myotonia	48		
Myotonia + paramyotonia	24		
Myotonia + progressive muscle weakness	18		
No myotonia	12		
No myotonia + progressive muscle weakness	4		
**Attack Triggers**			
Total respondents	91		
Female respondents	43		
Cold environments	69		
Rest after exercise	61		
Stress or fatigue	43		
Alcohol	41		
Hunger	39		
Changes in activity level	37		
Potassium in food	32		
Specific foods or beverages	32		
Changes in humidity	32		
Extra sleep	31		
Pregnancy	12		
Illness of any type	24		
Menstruation	8		
Medication	15		
Potassium supplements	13		
**Typical time of day of attack occurrence**			

Respondents	86		
Morning	48		
During sleep	39		
Upon waking	37		
Afternoon	25		
Evening	17		
**Prodromal symptoms**			
Respondents	70		
Day prior: positive response	27		
Immediately prior: positive responses	60		
**Attack frequency**			
Respondents	87		
1-3 per month	26		
1 per week	18		
2-6 per week	21		
**Typical attack duration**			
Respondents	80		
<5 min	8		
5-15 minutes	3		
15-30 minutes	11		
30 minute to an hour	17		
1-2 hours	5		
3-4 hours	5		
10-15 hours	2		
20 hours-1 day	4		
1-2 days	8		
>2 days	17		
**Longest attack**			
Respondents	83		
Over 1 week	19		
**Body region affected by attack**			

Respondents	92		
Calves	86		
Arms	84		
Hands	83		
Thighs	83		
Feet	70		
Eyelids	58		
Face	57		
Neck	54		
Speaking musculature	53		
Hips	39		
Breathing musculature	24		
Trunk	5		
**Severity of most attacks**			
Respondents	90		
Very mild – barely noticeable to others, few limitations	5		
Mild – have only some limitations on mobility, others would notice I am in an attack	39		
Moderate – have some movement, can self-medicate	32		
Severe – can speak, cannot move at all, can call for help	12		
Very severe – cannot speak, cannot call for help	2		
**Severity of interictal symptoms**			
Respondents	89		
Severe, impairs activities of daily living	11		
Moderate	12		
Mild	30		
No symptoms	36		
**Muscle pain**			
Respondents	92		
Muscle pain during attacks	38		
After attacks	57		
None	24		
**Frequency of attacks over time**	**Worsened**	**Stayed the same**	**Improved**

Childhood	64	6	0
Puberty/teenage years	31	5	3
20-39	12	7	4
40-69	9	3	3
70+	1	0	0
Menopause	1	0	2
**Muscle stiffness during attacks**	**Worsened**	**Stayed the same**	**Improved**
Childhood	40	7	0
Puberty/teenage years	14	6	3
20-41	5	5	5
40-71	6	3	2
70+	0	0	0
Menopause	2	0	1
**Muscle weakness during attacks**	**Worsened**	**Stayed the same**	**Improved**
Childhood	5	10	0
Puberty/teenage years	4	7	6
20-40	4	6	18
40-70	8	1	11
70+	1	0	0
Menopause	0	0	1
**Permanent muscle weakness**	**Worsened**	**Stayed the same**	**Improved**
Childhood	18	8	0
Puberty/teenage years	4	8	0
20-42	3	7	0
40-72	10	1	0
70+	1	1	0
Menopause	0	2	0
**Progressive myopathy (muscle dysfunction) or permanent weakness**			
Respondents	92		
Yes	28		
No	48		
Unsure	16		

**Level of Disease Control**			
Respondents	83		
Mostly controlled	37		
Needs improvement	42		
Optimal	4		
**Acute Medications**	**Mostly Controlled**	**Needs Improvement**	
Respondents	14	20	
None	2	4	
Hydrochlorothiazide	2	5	
Salbutamol	2	4	
Mexiletine	2	1	
Flecainide	2	0	
Furosemide	1	0	
Acetazolamide	1	4	
Magnesium	1	3	
Torasemide	1	0	
Fenoterol	1	0	
Orphenadrine	1	0	
Nonsteroidal anti-inflammatory drugs	1	1	
Calcium	1	1	
Carbamazepine	0	1	
Potassium	0	1	
Propafenone	0	1	
Muscle relaxants	0	1	
**Chronic Medications**	**Mostly Controlled**	**Needs improvement**	
Respondents	23	26	
Hydrochlorothiazide	5	4	
Mexiletine	5	1	
No treatment regimen	4	16	
Flecainide	3	0	
Acetazolamide	2	5	
Salbutamol	2	1	
Fludrocortisone	1	1	

Magnesium	1	1	
Torasemide	1	0	
Furosemide	1	1	
Omeprazole	1	0	
Vitamin C	1	0	
Limptar	1	0	
Salt	1	0	
Potassium	0	1	
Electrolyte fluid	0	1	
**Able to abort an attack**			
Respondents	86		
Never	18		
Occasionally	13		
**Episodes of weakness that improve with potassium intake**			
Respondents	63		
Positive	8		
**Years to arrive at current regimen**			
Respondents	22		
10+ years	12		
5-10 years	5		
1-5 years	6		
<1 year	2		
**Pregnancy**	**Worsened**	**Stayed the same**	**Improved**
Frequency of attacks over time	11	0	1
Muscle stiffness during attacks	6	0	2
Muscle weakness during attacks	1	0	4
Permanent muscle weakness	1	2	0
**Effects of surgery**			
Respondents	74		
Never had surgery	17		
Non-anesthetic surgical complications	13		

**Effects of local anesthesia**			
Respondents	83		
Never had local anesthesia	7		
Complications related to local anesthesia	8		
**Effects of general anesthesia**			
Respondents	86		
Never had general anesthesia	19		
Complications related to general anesthesia	20		
